# Structural Characterization and Functional Properties of Flaxseed Hydrocolloids and Their Application

**DOI:** 10.3390/foods11152304

**Published:** 2022-08-02

**Authors:** František Lorenc, Markéta Jarošová, Jan Bedrníček, Pavel Smetana, Jan Bárta

**Affiliations:** 1 Department of Food Biotechnologies and Agricultural Products’ Quality, Faculty of Agriculture and Technology, University of South Bohemia in České Budějovice, Studentská 1668, 370 05 Ceske Budejovice, Czech Republic; bedrnicek@fzt.jcu.cz (J.B.); smetana@fzt.jcu.cz (P.S.); 2 Department of Plant Production, Faculty of Agriculture and Technology, University of South Bohemia in České Budějovice, Na Sádkách 1780, 370 05 Ceske Budejovice, Czech Republic; jarosovam@fzt.jcu.cz (M.J.); barta@fzt.jcu.cz (J.B.)

**Keywords:** *Linum usitatissimum* L., flaxseed, food hydrocolloids, flaxseed proteins, flaxseed gum, functional properties, health benefits

## Abstract

Flaxseed is an excellent source of valuable nutrients and is also considered a functional food. There are two types of hydrocolloids in flaxseed: flaxseed gum and proteins. Flaxseed gum exhibits emulsifying and foaming activities or can be used as a thickening and gelling agent. Due to its form of soluble fiber, flaxseed gum is related to many health benefits. Flaxseed proteins have various functional properties based on their physicochemical properties. While albumins possess the emulsion-forming ability, globulins better serve as foaming agents. Flaxseed proteins may also serve as a source of functional peptides with interesting biological and health-related activities. Functional properties and health-related benefits predetermine the application of these hydrocolloids, mainly in the food industry or medicine. Although these properties of flaxseed hydrocolloids have been recently and extensively studied, they are still not widely used on the industrial scale compared to other popular plant gums and proteins. The aim of this review was to present, discuss and highlight the recent discoveries in the structural characteristics and functional and biological properties of these versatile hydrocolloids with respect to factors affecting their characteristics and offer new insights into their potential applications as comparable alternatives to the other natural hydrocolloids or as the sources of novel functional products.

## 1. Introduction

Flax (*Linum usitatissimum* L.) is a traditional crop grown to produce oilseeds or fiber. Flaxseed has been mainly used for oil production, whereas the remaining by-products in the form of defatted flaxseed meal (FM) or oil cake are usually fed to animals [1,2]. Edible flaxseed products include whole, ground, or crushed flaxseed, extracted oil, or gum [3]. Dietary flaxseed has recently been used in the human diet due to its nutritional value, determined mainly by the high-quality oil and proteins followed by the high content of insoluble and soluble dietary fiber [1]. Together with the high content of phytoestrogens in the form of lignans, these compounds of flaxseed are related to many health benefits [4]. Flaxseed fulfills the parameters of functional food due to its significant content of polysaccharides and proteins with a broad spectrum of functional properties [3]. However, for maximizing the potential of flaxseed in the human diet, its form appears to be a crucial factor. Compared to whole or crushed seeds, a better bioavailability of lignans from ground flaxseed has been reported [5], suggesting the influence of its form on the bioavailability of other involved bioactive compounds. Flaxseed is composed of 37–41% fat, 28–29% total dietary fiber, 20% protein, 6.5–7.7% moisture, and 2.4–3.4% ash [3,6]. Flaxseed oil has a balanced ratio of unsaturated fatty acids, mainly α-linolenic acid representing approximately 40–60% of flaxseed oil content (by traditional flax cultivars), followed by the oleic and linoleic acids [1]. Dietary fiber consists of soluble and insoluble fiber, whereas the prevailing fraction (60–80% of total fiber) represents insoluble fiber [7] composed of lignin, hemicellulose, and cellulose [8]. Regarding its chemical composition, flaxseed is a rich source of substances possessing hydrocolloidal properties.

Hydrocolloids are colloidal substances with an affinity for water, represented mainly by polysaccharides and proteins. Hydrocolloids produce viscous solutions, pseudo-gels, or gels in water and can be used to thicken and stabilize formulations. As an ingredient in foods, they influence viscosity and texture [9]. Most commercially important hydrocolloids are of natural origin. Plant sources of hydrocolloids, including popular gums and vegetable proteins, are extracted from trees (cellulose), tree gum exudates (gum arabic, gum karaya, gum ghatti, gum tragacanth), plants (starch, pectin, cellulose), seeds (guar gum, locust bean gum, tara gum, tamarind gum and soybean, pea and wheat proteins) or tubers (konjac mannan). The important hydrocolloids can also be found in red seaweeds (agar, carrageenan) and brown seaweeds (alginate). Lots of natural hydrocolloids have a microbial origin (xanthan gum, curdlan, dextran, gellan gum, cellulose) and animal origin (gelatin, caseinate, whey protein, egg white protein, chitosan) [10]. The natural gums may serve in foods mainly as stabilizers, thickening, gelling, and encapsulating agents [11]. Vegetable protein isolates can be used as foaming, emulsifying and gelling agents. Some of these proteins can also be characterized by their high water- and fat-holding capacity and dough formation [12]. Beyond that, novel functional products possessing enhanced functional properties can be obtained by processing the raw hydrocolloids, for instance, by enzymatic hydrolysis. Hydrolysis of proteins can lead to the production of functional peptides. Protein hydrolysates from rice [13] or rice bran [14] possess better functional, especially emulsifying, properties compared to native proteins. Hydrolysis of egg yolk improves its heat resistance and emulsifying properties [15]. Soy protein hydrolysates can show higher solubility, oil-holding, and foaming capacity than the original soy protein isolate [16]. Partially hydrolyzed guar gum shows improved rheological and pasting characteristics [17]. Oligosaccharides produced by enzymatic hydrolysis of guar gum [18,19], basil seed gum [20], and glucomannan gum [21] can be used as prebiotics. As a unique property, it was reported that new substances obtained by acid and enzymatic hydrolysis of ɩ-carrageenan might induce significant retardation of recrystallization in the sucrose solution [22]. Apart from food applications, hydrolysates of hydrocolloids often have various specific and direct effects on molecular mechanisms of human physiology [23]. Flaxseed represents a source of two further significant natural hydrocolloids—flaxseed gum (FG) and flaxseed proteins (FPs). The specific attributes of FG and FPs determine their functional properties, potentially usable in various applications, especially in the food industry and health-related applications.

FG is a heteropolysaccharide composed of neutral and acidic monosaccharides. Its presence is concentrated in the seed coat [24]; thus, it can be easily extracted by water from whole seeds, meals, or hulls of flaxseed [25]. Although hydrocolloids are mostly not appropriate emulsifiers [9], FG is a suitable additive for stabilizing water-oil emulsions. Its emulsifying activities are comparable to or better than commonly used emulsifiers [26]. Other functional properties of flaxseed gum are represented by its water- and oil-binding capacity [3,27], thickening, stabilizing [25], and gelling activities [28]. Due to its form of dietary fiber and presence of phenolic compounds, FG possesses antioxidants [24,29,30,31], anti-obesity [32], anti-cholesterol, and anti-diabetic activities [33].

FPs form approximately 20% of flaxseed weight [34]. The high quality of FPs is determined by the increased presence of essential amino acids, and their spectrum is comparable to the spectrum of soy protein [3]. The functional properties of FPs depend on their physicochemical characteristics. Flaxseed protein isolate (FPI) can be used as a foaming agent and emulsifier [35]. The mixtures of FG and FPs act better as an emulsifier, foaming, and water-binding agent than solely FG and FPI [36]. FPs can serve as a source for producing functional peptides with various biological activities related to health benefits, e.g., antioxidant and antimicrobial properties, anti-diabetic activities, or they act as angiotensin-converting enzyme inhibitors [37].

In this review, we aim to describe the chemical structures of flaxseed polysaccharides and proteins; present and discuss the functional properties of both types of hydrocolloids with respect to factors affecting their characteristics; summarize their recent application, and suggest their potential for future applications, especially in the food industry.

## 2. Flaxseed Gum

FG, also known as flaxseed mucilage, is present in the outer layer of the seed coat [38], and its content in the whole seed ranges between 3.5–15.0% [24,39,40], depending on the flax variety and the planting area [41]. The extraction method and its parameters determine the yield [39], purity, and properties of FG. For instance, the temperature of extraction is an essential factor affecting the chemical composition [29,41], functional properties [29,40,41] and biological activities [29]. Functional properties of FG can also be determined by the processing after extraction, for instance, by drying [42]. Thus, the applied extraction method and further processing are the key factors for obtaining FG with the optimal properties according to subsequent use.

### 2.1. Methods of Extraction

Polysaccharides are easily extractable from flaxseed; it is possible to obtain them from whole seeds [41], seed hulls [43], or oilseed cake [27]. Safdar et al. [30] used four methods for the extraction of FG from whole flaxseeds with differences in the relative yield of FG from flaxseeds: hot water extraction (9.0%), ultrasound-assisted extraction (7.8%), microwave-assisted extraction (7.0%) and alkaline–acidic extraction (6.4%). For the highest yield of FG, hot water extraction is preferable, whereas, for maximal purity, it is most suitable the ultrasound-assisted extraction method. Cui et al. [44] suggested the optimal condition of aqueous extraction with respect to yield, apparent viscosity, and protein content of the final gum extract. These conditions were a temperature of 85–90 °C, a pH 6.5–7.0 and a water:seed ratio of 13. Subsequently, Kaushik et al. [41] reported that the yield, purity of carbohydrates, and protein content could be significantly affected by the extraction temperature within the aqueous extraction. They observed the increasing yield of FG from 2.1 to 8.4% with increased extraction temperature from 30 to 90 °C. They have also suggested that the longer extraction time increases the yield of FG. Fabre et al. [45] reported ultrasound-assisted extraction as the most effective method with a 6.8% relative yield, followed by hot-water extraction (6.5%) and microwave-assisted extraction (2.1%). Except for the methods and conditions of extraction, the yield of FG can vary with the type of flax cultivar, crop age, and climate [46]. Since the extraction method or its conditions, such as temperature or length of extraction within the aqueous extraction, increase the yield of extracted FG or can change the chemical composition, as it is discussed in Section 2.2, too harsh conditions can cause the degradation of FG and formation of artifacts [46]. These changes could probably lead to the deterioration of the functional properties of FG and limit its potential applications. Thus, the effort to maximize FG yield should not be the only criteria for extracting FG.

### 2.2. Chemical Composition and Structure

FG composition depends on genotype, production practices, environment, and storage conditions [40]. The potential yield of FG is also determined, apart from the extraction conditions, by the cultivar of flax. The chemical composition of FG is shown in Table 1. FG is composed mainly of polysaccharides, up to 90% of relative content. Kaushik et al. [41] reported 80–90% saccharides content in FG, depending on the extraction temperature. Other studies present lower values for saccharides within FG, reaching only 68% [47] or 71% [39]. The minor part of FG represents proteins, lipids, ashes [24,41], and other compounds, such as phenolic acids [29]. However, within several studies describing the chemical composition of FG, the different values reported for saccharides, proteins, and ashes determine the wide range of their relative content in FG (Table 1). The content of lipids is low compared to other constituents. The relative content of saccharides and proteins depends on the temperature of extraction, whereas increasing the extraction temperature from 50 °C to 90 °C reduces the polysaccharides yield (90.3% → 80.0%) but increases the content of proteins (4.7% → 15.1%) in FG. Conversely, there were not reported significant changes in the content of ashes and fats induced by the changes in the extraction temperature [41].

FG has a highly branched polymeric structure [48]. Polysaccharidic complexity is determined by the presence of polysaccharidic fractions with various molecular weights (MW) composed of monosaccharides. According to the literature, the presence and abundance of monosaccharides can be quite variable, as shown in Table 2, and significantly affect the properties of FG. This diversity can be determined by various factors, such as the temperature of aqueous extraction [41] and flax cultivar [49]. There was described the presence of the neutral (arabinoxylan-rich—MW up to 5500 kDa), acidic (rhamnogalacturonan–I rich—MW < 500 kDa) and composite arabinoxylan-rhamnogalacturonan I-rich (1700 < MW < 700 kDa) fractions [50]. Warr et al. [51] suggest that the neutral fraction accounts for about 75% of flaxseed polysaccharides, and the remaining acidic fraction makes up about 25%. Hellebois et al. [50] reported broad MW distribution with the dispersity index (Ɖ) ranging from 2.11 to 3.82, whereas this parameter was not dependent on the extraction method or genotype of flax. They identified four polysaccharide populations corresponding to 4513 (<3%), 1627 (31–48%), 701 (22–43%) and 231 (19–35%) kDa. Qian et al. [43] assessed the MW of polysaccharides within FG composed of high MW arabinoxylans (1470 kDa) and rhamnogalacturonans with a higher MW fraction (1510 kDa) and lower MW fraction (341 kDa). Safdar et al. [24] reported the average MW of FG as 1322 kDa with a polydispersity ratio of 1.6 for Mw/Mn and 2.4 for Mn/Mz.

**Table 1:** Chemical composition of flaxseed gum powder. Presented as the ranges of values reported in the literature (on wet matter basis).

Constituent	Content [g/100 g]	References
Proteins	1.5–22.1	[24,39,41,47,52]
Ashes	0.6–11.2	[24,39,41,47]
Saccharides	67.7–90.4	[24,41]
Fats	0.3–2.1	[24,41,47]
Moisture	3.4–5.5	[24,41,47]

A neutral polysaccharide is an arabinoxylan consisting of L-arabinose, D-xylose, and D-galactose [24]. Warrand et al. [53] described the structure of neutral polysaccharides as the 1,4-linked β-D-xylans substituted by D-arabinose or terminal L-fucose, D-xylose, or D-galactose, whereas they vary in galactose and fucose residues within their side chains. β-1,4-linked xylose backbones of arabinoxylans of FG are unsubstituted or substituted at O-2 and/or O-3 positions by 1-3 sugar residues [54]. Kaushik et al. [41] presented arabinose and xylose as the major monosaccharides of arabinoxylan, having a ratio ranging from 0.6 to 0.7, which did not vary with the extraction temperature. They have suggested that this ratio is responsible for a moderately branched structure of FG.

The acidic polysaccharide is represented by rhamnogalacturonan containing L-rhamnose, L-fucose, L-galactose, and D-galacturonic acid [24]. Qian et al. [43] described the structure of the acidic polysaccharide fraction of FG composed of a backbone consisting of rhamnogalacturonan-I interrupted by the small amount of homorhamnan or homogalacturonan. They also reported a high amount of monogalactosyl branches 3-linked to half of the 1,2-linked rhamnosyl residues. Another study reported that rhamnogalacturonan blocks could be monosubstituted at the O-3 position of rhamnose by side chains of terminal galactose or fucose, occasionally by neutral monosaccharides. The rhamnose/galacturonic acid content was similar since their calculated ratio was 1.22 to 0.85. A branching degree was estimated at 0.33 to 0.65 [55]. Authors also suggested that the variability of acidic fractions of FG is not related to the rhamnogalacturonan-I backbone but rather by the type and degree of substitutions. A significant contribution to the relative abundance of acidic polysaccharides also determines the presence of galacturonic acid. There was reported only a minor content of galacturonic acids, approximately 7% [24]. However, other studies describe higher values, such as 23% [56], respectively 21–25% [49]. One of the former studies has described a high galacturonic acid content (approx. 40%), making it even the most abundant monosaccharidic constituent [57].

**Table 2:** Monosaccharide composition of flaxseed gum.

Monosaccharide	Relative Monosaccharide Compositions [%]			
	Safdar et al. [24]	Kaushik et al. ^a^ [41]	Qian et al. [56]	Cui and Mazza ^b^ [49]
Arabinose	8.3	25.4–28.0	9.8	6.9–10.7
Xylose	13.5	42.9–47.6	29.7	16.1–29.5
Galactose	18.7	13.0–14.0	17.2	15.8–21.6
Glucose	20.0	2.3–3.1	2.1	1.7–6.2
Rhamnose	23.9	7.0–8.2	12.7	16.7–20.4
Fucose	8.0	2.9–3.8	5.4	4.0–5.3
Galacturonic acid	6.8	ND	23.0	21.0–25.1
Mannose	0.4	NR	NR	ND
Ribose	0.2	NR	NR	NR
Glucuronic acid	0.03	NR	NR	NR
Glucosamine	0.2	NR	NR	NR

NR: not reported; ND: not detected; ^a^: extraction of flaxseed gum was performed at 30, 50, 70 and 90 °C, the range of the values is determined by the lowest and the highest values obtained within the extraction by different temperatures; ^b^: for the study were used three flax cultivars—NorMan, Omega and Foster, the range of the values is determined by the lowest and the highest value obtained within single cultivars.

The extraction conditions may affect the ratio between the types of monosaccharides in obtained FG. Kaushik et al. [41] reported decreased ratio of neutral:acidic monosaccharides from 6.7:1 to 5.7:1 as the extraction temperature was increased from 30 to 90 °C. Troshchynska et al. [58] found that the FG of brown seed cultivars Libra Bio and Recital were more acidic (pH 5.4–5.5) compared to the yellow-seeded cultivars Amon and Raciol (pH 5.8–6.1). However, Cui and Mazza [49] reported the lower content of acidic monosaccharides (rhamnose, fucose, galactose, and galacturonic acid) and, conversely, higher content of neutral xylose within brown-seed cultivar NorMan compared to yellow-seeded cultivars Omega and Foster. Thus, the relation between cultivar type and monosaccharidic composition of FG remains unexplained.

The extraction method significantly affects the yield of polysaccharides and proteins extracted from FG. Higher content of proteins and browning in color was observed within FG extracted at higher temperatures [56]. Moczkowska et al. [52] assessed 22% of protein and only 42% of polysaccharides content extracted by the alkaline method, whereas using the enzymatic-assisted method with ultrasound allowed for the extraction of 8% of protein and 69% of polysaccharides from FG. Furthermore, microstructural differences of FG extracted from six Chinese and Mongolian flax varieties were described [59]. Differences in the structures were also found between seven Italian flax cultivars. They also differed in chemical, physicochemical, and functional properties [46]. According to the studies, it is evident that the extraction conditions have a crucial role in the monosaccharide composition of FG, mainly determined by the ratio of neutral to acidic polysaccharides in FG. This ratio then significantly determines the physicochemical properties of FG, e.g., viscosity, and thus, also other functional properties [26].

### 2.3. Functional Properties

As reported in the previous chapter, the spectrum, and concentration of FG constituents, especially the carbohydrate composition, strongly affect its functional properties and potential applications that may be quite broad, as shown in Table 3. As a soluble fiber, FG has a significant ability to bind water, reaching values of 16–30 g water/1 g FG [3]. FG is also responsible for the high-water holding capacity of flaxseed flour, reaching approximately 4.15 g of water/1 g of flaxseed flour [60]. The ability of FG to bind oil is approximately 1 g oil/1 g FG [27]. FG is generally characterized by high viscosity (rheological properties or parameters such as viscosity, shear-thinning, and Newtonian flow, as the physical characteristics, are in this review involved within functional properties of FG due to their influence on the functioning of FG within certain applications) comparable to acacia gum and higher than guar and tamarind gums [61]. A higher proportion of neutral polysaccharides (arabinoxylans), which have a higher MW, increases viscosity and exhibits the shear-thinning flow behavior of FG. Conversely, as the abundance of lower MW acid polysaccharides increases, the viscosity decreases, and Newtonian flow behavior is exhibited. Viscosity and fluidity are also affected by the pH of FG. The lowest viscosity of FG appears at pH 2. With increasing pH, the viscosity increases up to pH 8, when the viscosity is 3-times higher than FG with pH 2. However, at a pH higher than 8, the viscosity decreases again. Different varieties exhibited wide variations in viscoelastic properties of extracted FG. At the same concentration in solution (1 to 3%), it may have a form of viscoelastic fluid or a real gel [26]. The ratio of polysaccharides also affects the related rheological properties of FG, which may form a weak gel with a higher proportion of neutral polysaccharides. At high concentrations of acidic polysaccharides, FG appears as a viscoelastic fluid [62]. The addition of FG at the concentration of 0.08% to carrageenan gel within a fixed 1% concentration of polysaccharides in the solution decreased the syneresis from 11.0% to 6.6% of FG/carrageenan gel and increased the viscosity [63]. In their research, Hu et al. [40] reported that FG extracted at a temperature of 70 °C had a higher viscosity compared to FG extracted at 98 °C (96.7 vs. 78.8 mPa·s) on the first day after extraction but lower viscosity than 98 °C (70.1 vs. 71.9 mPa·s) after storage at 4 °C for eight days. FG extracted at 98 °C had a better foaming capacity (~135 vs. ~127%) and foam stability (~88 vs. ~79%) on the first day after the extraction, but after eight days of storage, these parameters stayed comparable for both FG (foaming capacity: ~132–133%, foam stability: ~86–87%). Emulsion capacity (~43–46%) and stability (~98–100%) were comparable for both types of FGs after the first day after extraction but decreased for FG extracted at 70 °C and stayed quite constant for FG extracted at 98 °C after storage for eight days. These observations indicate changes in the functional properties of FG extracted at different temperatures by hot water extraction.

The relation between emulsion stability and pH value was observed, where the highest stability of the emulsion, in the form of model salad dressing, was observed at pH 6. FG is a comparable or better emulsifier than Tween 80, gum arabic, and gum tragacanth. An FG concentration ranging from 0.5-1.5% is suitable for stabilizing water-oil emulsions [26]. High creaming stability (creaming volume: 100%) within emulsion containing 10% olive oil (*w*/*w*) was observed for the emulsion with a high content of FG (0.4%–0.5% *w*/*w*), as well as better rheological properties of the emulsion with same addition of FG [64]. FG shows similar or even better foam stability (~41%, 1.0 *w*/*w*) than gum arabic and xanthan gum (15–27%, 0.5 *w*/*w*) [42]. Significant functional properties of FG can be affected by the extraction temperature. It has been found that the viscosity and elasticity of FG decrease with increasing temperature and is also affected by the pH values of the FG solution [29]. Fabre et al. [45] reported that ultrasound-assisted extraction decreases the intrinsic viscosity of FG extracted by hot water from 12.5 dL/g to 6.2 dL/g, and the MW of the largest polysaccharides decreased from 1500 kDa to 500 kDa. Another study reported that FG extracted at 30 °C exhibited a water absorption capacity of 25.9 g/g, comparable to that of guar gum (22 g/g). However, there was observed a deterioration in emulsifying activity (emulsion activity index: ~150→~60 m^2^/g, 70→90 °C) and water absorption capacity (down to ~12 g/g, 90 °C) within FG extracted at higher temperatures. This study also stated that emulsifying activity can be affected by the content and composition of proteins in FG. Apart from that, FG extracted at higher temperatures exhibited an insignificantly increased fat absorption capacity [41].

The microstructure of FG also affects its functional properties and may vary between flax varieties [46,59]. Temperature stability is one of the properties determined by variety or its type. FG extracted from a yellow flaxseed cultivar showed higher temperature stability than FG extracted from brown flaxseeds. FG of yellow flaxseed also showed higher MW (1150–1340 kDa), higher intrinsic viscosity (6.63–5.13 dL/g), as well as viscoelastic and thickening properties [50]. The functional properties such as viscoelasticity, gelling ability, and emulsifying activity can also be influenced by phenolic compounds, especially lignans and phenolic acids, and their migration within FG. An improvement in rheological and emulsifying properties was observed when the phenolic compounds were removed, which may be caused by changes in the spatial conformation of the protein or disruption of noncovalent interactions between phenolic compounds and polysaccharide chains [65]. Unfortunately, the natural structure of FG could have inherent problems associated with its use, including uncontrolled hydration rates, solubility dependent on pH, thickening, a drop in viscosity in storage, and probable microbial contamination. Thus, the authors report that the original structure of FG can be modified by crosslinking or esterification to obtain the desired and defined properties [25].

Based on the reported findings, it is evident that the genotype and extraction conditions, determining the chemical composition and structure of FG, significantly influence the functional properties of FG, and they are crucial to obtaining FG with the desired properties. Further processing of FG, such as drying (by spray, freeze, vacuum, or oven) or ethanol precipitation, also affects some functional properties of FG, such as emulsifying activity, foaming, and gelling ability [42].

### 2.4. Biological and Physiological Activities

In addition to the functional properties, FG also shows several biological activities. FG possesses strong antioxidant activity, which has been confirmed in several studies [24,29,30,31,66]. The antioxidant activity of FG is significantly affected by the presence of some phenolic compounds easily extractable with FG, such as caffeic acid, p-coumaric acid, epicatechin, ellagic, cinnamic, and vanillic acids. Similarly, like the polysaccharides and proteins of FG, it was found that the content and composition of phenolic acids and related antioxidant activities are affected by the extraction temperature. With increasing extraction temperature (25 → 40 → 60 °C), the scavenging activity against DPPH (2,2-diphenyl-1-picrylhydrazyl) radical (4.4 → 12.3 → 29.6% radical scavenging activity) and the total content of polyphenols (12.4 → 13.0 → 18.6 mg GAE/100 g) increased. However, the content of caffeic acid (6.6 → 6.4 → 6.1 mg/L) and *p*-coumaric acid quantified together with epicatechin (1.6 → 1.4 → 1.4 mg/L) decreased slightly with increasing temperature, whereas the cinnamic acid content remained unchanged (2.3 mg/L) at all temperatures in FG solution. On the other hand, the ellagic acid content increased significantly at 60 °C (1.2 → 1.1 → 3.1 mg/L), and vanillic acid was detected only in FG extracted at 60 °C (5.4 mg/L) and was nearly comparable to the content of caffeic acid [29]. Although flaxseed contains high amounts of lignans (as mentioned in Section 1), it remains a question whether these compounds are extracted together with FG and how much they participate in the antioxidant activity of FG.

Safdar et al. [24] report a high antioxidant potential of FG and a rise in antioxidant activity due to increasing FG concentration in an aqueous solution. The highest antioxidant activity was found at the highest measured concentration (30%) of FG expressed as total antioxidant activity [55], reaching the values of approx. 588 µg Trolox/1 mL FG. The scavenging activities reached 98% against the radical DPPH and 72% against ABTS (2,2′-azino-bis(3-ethylbenzothiazoline-6-sulfonic acid). Bouaziz et al. [31] previously assessed the antioxidant properties of FG extracted from flaxseed. They reported higher scavenging activity against DPPH (expressed as the half inhibition concentration = IC_50_) for FG (IC_50_ = 2.5 mg/mL) compared to the antioxidant activity of polysaccharides of guara fruits (IC_50_ = 10.8 mg/mL), prickly pear peels (IC_50_ = 10.8 mg/mL) and almond juice processing by-products (IC_50_ = 2.87 mg/mL) but was lower than for polysaccharides of garlic straw (IC_50_ = 0.74 mg/mL) and pistachio juice processing by-products (IC_50_ = 1.61 mg/mL). Yang et al. [66] compared the antioxidant activities of total FG and flax oligosaccharides extracted from FG by enzymatic degradation. The purified mixture of oligosaccharides from FG exhibited better antioxidant activities, expressed by scavenging of DPPH, ABTS and hydroxyl radicals, compared to native FG. FG can also potentially contribute to antimicrobial and antioxidant properties of FG-containing coatings and films, whose applications are described in Section 2.5.

Similarly, like other types of soluble fiber, FG forms a viscous or gel-like material when dissolved in water [67]. FG affects digestion in more ways. It increases food weight in the gastrointestinal tract and feces [3]. It presumably interacts with fats and sugars in the intestine and inhibits their absorption. This hypothesis is supported because fats and sugars are excreted as part of the feces when FG is consumed to a greater extent than in the case of subjects that do not ingest FG [68]. Consumption of FG also causes the prolonged feeling of satiety, accompanied by more extended inhibition of the enzyme ghrelin—a signal digestive peptide that stimulates hunger [68]. Reductions in body weight and body fat, respectively, and total triglycerides, have been confirmed in animal clinical trials due to reduced fat and energy absorption within animal subjects. This phenomenon has probably been related to the regulation of intestinal microflora as an effect of FG consumption. In particular, the inhibition of *Clostridium* bacteria from the phylum of Firmicutes was observed. Conversely, they report a slightly increased presence of bacteria from the phylum Bacteroidetes and a strong increase in the occurrence of strains from the phylum Proteobacteria [32].

### 2.5. Potential Applications

FG is a substance without taste properties, which is an essential property for its use in the food industry [46]. However, it was reported that adding FG may improve the sensory characteristics (appearance, structure and porosity, crumb color, smell, and taste) of freshly baked gluten-free bread when 1.8% or 2.4% of starch is substituted by flaxseed gum [69]. FG can replace commonly applied thickeners, emulsifiers, beverage stabilizers, and similar applications. Its presence in bread affects the baking properties, such as stickiness, rheology of dough, and baking process. In addition, it improves the texture, which is softer, makes the bread soft for longer and delays hardening during storage, probably caused by the presence of the arabinoxylan fraction. It has been suggested that 0.5% of FG (flour basis) could replace 0.1% xanthan or guar gums. FG positively affects the gluten behavior in the dough since an increase in volume was observed during leavening and baking. In bakery products and ice cream, FG can also substitute egg whites [26].

The complex viscosity of salad dressing may increase with the rising concentration of FG from 0.13 Pa·s (0% FG) to 6.61 Pa·s (0.75% FG) to finally 37.2 Pa·s (1.5% FG) and prevent flocculation and coalescence of the contained oil. The most stable emulsion was observed after the addition of 0.75% (*w*/*w*) of FG and 2.5% (*w*/*w*) of salt at pH 4 [70]. As an emulsifier, FG can also be used as a part of beverages. It can suppress creaming and increase the viscosity of fruit or vegetable juices since this phenomenon has been observed in the case of unfiltered carrot juice. At the same time, adding 0.5 g/kg of FG was most effective for stabilizing the cloudy carrot juice [71]. FG effectively improves texture characteristics and reduces the syneresis of stirred yogurt containing 0.6 g of FG per 100 mL of yogurt (14.3 → 0.1 mL). It also supports the optimal growth of starter microorganisms [72]. The 0.1, 0.2, or 0.3% concentration of FG in the heat-induced gel increased the water-holding capacity by 7.0%, 15.5%, and 25.8%, respectively, thus offering the use of FG within meat products [73]. FG, up to a content of 12%, in a mixture of carrageenan and gellan gum improved the sausage texture and color of the sausage. FG enhanced the hardness, springiness, and emulsion stability of the product [74]. In addition to the possibilities of using FG itself, it also has potential in interactions with proteins, and the possibility of applications of the complexes of these components is also studied. These heterogeneous mixtures may exhibit a combination of not only functional properties of the two components separately but also properties determined by their interaction.

A study focused on flax–protein interactions observed the formation of electrostatic coacervates consisting of whey protein and FG. These biopolymers were characterized by high viscosity and viscoelasticity, predetermining their use as a texture modifying component of food products. The maximum coacervate formation and viscosity were assessed when the mixture of whey protein isolate/flaxseed gum was prepared in water at pH 3.8, constant biopolymers concentration of 0.05% (*w*/*w*), and their ratio of 2:1 (*w*/*w*) [75]. Coacervates of FG and FPs were used as coating materials to encapsulate unstable or volatile compounds. Kaushik et al. [76] used FG and FPI crosslinked with glutaraldehyde to encapsulate flaxseed oil with three core (oil)-to-wall ratios (1:2, 1:3, and 1:4) to preserve its oxidative stability. The highest microencapsulation efficiency (87%) was observed at the spray-dried microcapsules with a surface oil of 2.8% at a core-to-wall ratio of 1:4 and an oil load of 20%. Pham et al. [77] enriched FPs-FG complex coacervates, encapsulating flaxseed oil, with flaxseed polyphenols and hydroxytyrosol to enhance the efficiency of this material against oxidation of flaxseed oil which was better than for ordinary FGI/FPI complex coacervates. The optimal protein-to-gum ratio was 6.00. The microcapsules with the lowest surface oil (1%, *w*/*w*) and highest microencapsulation efficiency (95.4%) were produced using (FPI-hydroxytyrosol)/FG complex coacervates. FG and rice bran protein coacervates were also successfully used to encapsulate vanillin to improve its thermostability and shelf-life. The optimum ratio and total concentration of rice bran protein and FG, for the maximum strength of the complex coacervate, were 9:1 and 0.4, respectively [78].

Lai et al. [79] reported the protective capacity of FG towards *Lactobacillus rhamnosus* during co-extrusion microencapsulation, where the optimized parameters for microencapsulation of the bacterium were 1.0 mL/min core flow rate, 0.4% (*w*/*v*) chitosan coating and 0.4% (*w*/*v*) FG. The prebiotic effect of FG improved the survivability of *L. rhamnosus* in the gastrointestinal tract. They also reported the partial digestion of FG during gastrointestinal transit and the promoted growth of the *L. rhamnosus* [80]. FG had successfully protected *L. rhamnosus* from the harsh environment when it was microencapsulated in hawthorn berry tea, where the minimum requirement of 10^6^–10^7^ CFU/mL of probiotic cells was assessed to exert therapeutic health effects [81]. There was a determined high resistance of FG against hydrolysis by acid (1.5%) and pancreatin (2.6%) and a 98% prebiotic score, which is higher than commercial prebiotics including inulin, fructooligosaccharides, and isomaltooligosaccharides. The optimal concentration of FG in cultivation media, promoting the best in vitro growth of *L. rhamnosus* for 36 h, was 0.8%. Due to the prebiotic capacity of flaxseed mucilage and its symbiotic relationship with a probiotic bacterium, authors have also suggested the potential incorporation of optimized *L. rhamnosus* microbeads for developing other functional foods [80].

FG has been successfully used as a functional agent in edible coatings or films, protecting plant or food products against microbial spoilage and oxidative deterioration, thus preserving their sensory characteristics and safety. FG-based coatings preserved the sensory attributes, especially color, of cheese [82] and inhibited the invasion of foreign microorganisms (*Escherichia coli* and *Staphylococcus aureus*) [83]. As a part of edible coatings, FG can be used to prolong shelf-life and preserve the microbiological quality and sensory parameters of fresh fruits. The coatings containing 0.6% of FG and 500/800 ppm of essential lemongrass oil had promising effects on the sensory attributes and other overall quality parameters of ready-to-eat pomegranate arils [84]. Coating of FG with chitosan applied on pieces of fresh cantaloupe preserved sensory characteristics and increased the consumers’ acceptance of the fresh-cut fruit, stored at 4 °C for 12–15 days [85]. FG combined with other polysaccharides (chitosan, pullulan, nopal, and aloe mucilages), used for a coating of fresh-cut pineapple pieces, improved the quality and prolonged the shelf-life of the fruit for 6 days compared to control samples [86]. FG was also used as a constituent of coating for yacon as the carrier of the probiotic bacteria *Lactobacillus casei,* preserving the number of viable cells throughout the storage at 8 log CFU g^−1^ [87].

Apart from the food industry, there is potential to use these mixtures in the medicine, pharmaceutical, and cosmetic industries [25]. The biological properties of FG may have a positive influence on human health, predetermining its use in the treatment of diabetes, cholesterol reduction [33], the development of obesity [32], and it can have a role in the management of hyperglycemia [88]. FG also has potential for non-food and non-health applications, such as in the printing, textile, tobacco, and paper industries [26]. In immobilized form, FG can be used to produce gel particles enabling ecological adsorption of oils from wastewater, while the adsorption properties of these particles overcome the effect of activated carbon [89]. It can also be used in the mining industry. The ability of FG in the flotation of fluorite from calcite has been demonstrated [90]. Together with cellulose nanocrystals, FG may form nanocomposite materials or biopolymers that can be used to produce bioplastics [91].

As reported in many studies, FG possesses similar properties to other plant gums, so they can successfully substitute in various food products. However, FG protein coacervates may become highly functional agents that can potentially replace commercial gums in the future. Further research should also be focused on the potential application of FG related to their unique biological properties, such as the development of new types of functional coatings to protect not only sensitive foods but also various natural products and preserve their native properties. FG may become an active constituent of encapsulating material which would serve for the transportation and protection of probiotics or drugs and other bioactive compounds within the human gastrointestinal tract to the intestines avoiding the harsh condition of the stomach. Like the other plant gums, especially gum guar, FG can be processed by hydrolysis or other treatment to produce functional and health-beneficial oligosaccharides usable in food products or pharmaceuticals. Thus, there are many directions and possibilities for future research related to FG within the food and pharmaceutical industry.

**Table 3:** Functional properties of flaxseed gum and its potential applications in food products.

Functional Properties	Potential Application and Effects/Types of Food Products	References
Viscosity	Increase viscosity of food products or beverages; affects rheological properties/fruit and vegetable juices, salad dressings, bakery products	[26,40,61,70,71]
Shear thinning and gelling properties	Increase viscosity or gelling properties of food products/gelled food products	[62,63,67]
Water holding and oil binding capacities	Texture improvement; increase in the water-holding capacity of meat products; improvement of appearance, structure and porosity of bread/meat and bakery products	[3,27,41,60,69]
Emulsifying activity and emulsion stability	Improvement of firmness and elasticity; stabilization of oil and water emulsions; egg white substitution/sausages, bakery products, ice creams	[26,64,74]
Stabilizing and thickening properties	Improvement of creaming stability; foaming stability; decrease in a syneresis rate; affects pasting, dough rheology and baking procedure/plant oil, stirred yogurts, bread or other bakery products	[26,42,63,64,72]
Thermal stability	Functional agent in heat-processed food products/thermal-treated food products	[50]
Coating properties	Coating of plant or food products leading to prolonged shelf-life of food products due to inhibition of microbial spoilage and preserving the sensory characteristics; encapsulation of unstable compounds protecting them against oxidative deterioration/fruits, cheese, oils, volatile compounds and other plant or food products	[76,77,78,82,83,84,85,86,87]
Prebiotic capacity	Promotion of survivability and growth of *Lactobacillus rhamnosus* in gastrointestinal tract/fruit tea, other functional food products	[79,80,81]
Sensory-affecting properties	Improvement of the sensory properties/bakery or other food products	[46,69]

## 3. Flaxseed Proteins and Peptides

FPs possess a high nutritional quality mainly determined by the amino acid composition and is comparable to the quality of soy protein [3]. In addition to the high nutritional value, flaxseed proteins and peptides provide interesting functional properties and biological activities.

### 3.1. Methods of Extraction and Purification 

Most of the methods applicable for extracting FPs were developed in the mid- and late-20th century. Nevertheless, the methods are currently still modified to achieve the highest possible yield, purity, and functionality of proteins, if needed. One of the original methods for extraction of FPs was performed via the salting-out procedure followed by dialysis. However, the yield of proteins extracted by this method was only 44% of proteins contained in flour from dehulled flaxseed [92]. Later, soaking of flaxseeds in 1% HCl for 16 h, followed by washing in 1% HCl and water, allowed the extraction of 56% of proteins from demucilaged and defatted flaxseed flour [34]. Alkaline extraction can be used to prepare a supernatant with dissolved FP, followed by the isoelectric precipitation of certain protein fractions. Sosulski and Bakal [93] precipitated 77% of FPs from alkaline extract (pH 10) by isoelectric precipitation at pH 4.5. This process allowed us to obtain FPI containing 92% of proteins, respectively 61% in flaxseed protein concentrate (FPC) [94]. Nwachukwu and Aluko [35] isolated flaxseed albumin and globulin from FM using NaCl for 1 h at room temperature. Perreault et al. [95] extracted FPs at pH 5.0 and used cellulase to hydrolyze the fibers. For extraction, another original extraction method can be used using buffer systems, mainly phosphate buffer, combined with alkaline or acidic precipitation [96,97]. After extraction by phosphate buffer, FPs can be separated into three fractions by size exclusion column Sephadex [98] or Sepharose [34]. After salting-out precipitation, it has been possible to separate FPs into three fractions [99]. It was also reported the separation of FPs into four fractions by anion exchange column DEAD-Sephadex [99]. Kaushik et al. [100] used Tris buffer (pH 8.6) followed by acidic precipitation at pH 4.2 to extract protein from defatted FM. FPs can also be isolated using hexametaphosphate [101]. The alkaline extraction at pH 8.0–9.5 followed by the acidic precipitation at pH 3.8–4.2 seems to be a very effective procedure for obtaining FPI and FPC from demucilaged and defatted FM with previously reported yields ranging from 51.1% to 93.7% [102]. The higher pH values up to 11.0 can be potentially used to increase the yield of FPs. However, Ye et al. [102] suggest using the preferred pH values, pH 7.0–9.0 instead of 8.5–11.0, due to the undesirable modification of proteins and the unpredictable reactions at extreme alkaline conditions, which may consequently negatively affects functional properties.

FPs can serve as a source of bioactive peptides in the form of FPs hydrolysates which are produced mainly by proteolysis using non-commercial or commercial proteases, such as Alcalase^®^, thermolysin, trypsin, pepsin, papain, pancreatin, and ficin [103]; Neutrase^®^, Protamex^®^ [104]; chymotrypsin, thermoase, elastase, and carboxypeptidase A/B or the combination of listed enzymes [37].

### 3.2. Structural Characteristics 

Flaxseed proteins (N × 6.25) represent about 20% of the flaxseed weight [3,6]. Nevertheless, the protein content observed in some Canadian cultivars can reach above 36% (N × 6.5) [34]. Morris [6] reported a relatively significant difference in protein (N × 6.25) content between brown-seeded (22.3 g/100 g seed) and yellow-seeded (29.2 g/100 g seed) varieties. The nitrogen content can be affected by applying a nitrogen fertilizer during growth, and it can also affect the oil content [34].

FPs have a balanced ratio of amino acids. The most abundant one is glutamic acid (20% *w*/*w*), followed at a distance by aspartic acid, arginine, and leucine (Table 4) since the high contents of these amino acids are comparable to the amino acid profile of soybean seeds [34]. Conversely, the content of the essential amino acid lysine is limiting [7]. The amino acid content values presented in Table 4 indicate only minor variability of amino acid content between different cultivars. Moreover, the assessed content of amino acids within FPs remains stable after the heat processing of seeds, for example, in extruded products from FM [105].

Globulin (linin) and albumin (conlinin) represent the prevailing fractions of FP. In the flaxseeds, there are also other minor proteins, e.g., hirudine, oleosin, prolamin, and glutelin [106]. However, the four latest mentioned proteins are minor in flaxseed and thus are not discussed in this review. Former studies report a different content of dominant globulins ranging from 58% [34] to 66% [107] of protein content. Nwachukwu and Aluko [35] described differences in amino acid composition between the globulin and albumin fractions. Flaxseed globulin is a rich source of sulfur-containing amino acids (methionine, cysteine) and branched-chain amino acids (valine, leucine, and isoleucine). The presence of other amino acids is lower but in balance [37]. Globulin contains primarily hydrophobic amino acids, while the albumin fraction is represented more by hydrophilic amino acids.

Flaxseed globulins belong to groups 11S or 12S seed storage globulins formed by high MW (252–298 kDa) proteins [37] composed of multiple 10–50 kDa polypeptide chains [35]. Formerly, Marcone et al. [108] assessed the total MW of linin by native-PAGE (polyacrylamide gel electrophoresis) for 535 kDa. Madhusudhan and Singh [107] determined the secondary structure of flaxseed globulin using the circular dichroism method. It contains 3% of the α-helix structure and 17% of β-structure, indicating its relatively disordered structure. Linin was separated by SDS-PAGE (sodium dodecyl sulfate-PAGE) into five subunits of different MW connected by disulfide bridges (11, 18, 29, 42 and 61 kDa). Six subunits (55, 54, 50, 45, 43, and 41 kDa) were identified by urea-PAGE. The 55, 54 and 50 kDa subunits were then separated via denaturation by 2-mercaptoethanol into one 20 kDa basic subunit and 40 kD acidic subunit. Marcone et al. [108] identified the major subunit after purification by gel-filtration chromatography with a size of 320 kDa. SDS-PAGE revealed five globulin subunits of sizes 14, 25, 30, 35, and 51 kDa. In a more recent study, a large 365 kDa fraction was isolated from defatted and dehulled flaxseed from the cultivar NorMan by anion exchange chromatography, where 20, 23, and 31 kDa subunits were detected by SDS-PAGE [109]. Krause et al. [110] identified the subunits with a similar MW, specifically the protein bands of 21, 36, and 54 kDa belonging to 7S globulin.

Flaxseed albumin (conlinin) belongs to the group of 2S albumins and represents 20–42% of the total flaxseed proteins [34,97]. Madhusudhan and Singh [111] isolated conlinin from FM and reported its structure in a single polypeptide chain with an MW of 16–18 kDa. Nwachukwu and Aluko [35] reported a lower MW (10 kDa) of conlinin. The primary structure of conlinin contains 168 or 169 amino acids [37]. It has a more ordered structure than globulin, consisting of 26% α-helix type structure and 32% β-structure, and its amino acid composition shows a higher content of lysine, arginine, cysteine, and glutamate [111]. Liu et al. [112] describe conlinin as the major protein associated with FG and emphasize its essential role in the functional properties of FG.

### 3.3. Functional Properties

Similarly, like FG, FPs also provide interesting functional properties applicable mainly in the food industry, as presented in Table 5. The variances in the structure and physicochemical properties of flaxseed albumin and globulin induce different functional properties of each protein fraction. One of these properties is a different affinity of albumins and globulins for water. The proteins of the globulin fraction, which are poorly soluble in water, show a higher foaming capacity. On the other side, hydrophilic albumin provides better emulsion-forming ability [35]. The functional properties of FPC are also affected by their production methods. The alkaline and enzymatic extraction are suitable for the FPC applied in emulsion-based foods. On the other hand, enzymatic-solvent extraction is more applicable for producing a good source of protein for a food system [113].

FPs and their isolates are less soluble than proteins present in the seeds of other oilseed crops due to the significant content of globulins. FPI can exhibit relatively high thermal stability and higher emulsion activity index (375.51 m^2^/g), stability index (179.5 h), and zeta potential (−67.4 mV) than whey and soy protein isolate, sodium caseinate and gelatin. Water binding capacity (~4.0 g/g) was higher than for whey protein isolate, sodium caseinate, and gelatin and was comparable to soy protein isolate. The fat binding capacity (~2.7 g/g) was higher than in the case of soy protein isolate and gelatin and comparable to whey protein isolate and sodium caseinate. FPs can also stabilize emulsions at a low pH [100]. As a result, emulsions containing FPs are more stable and better absorbable in a strongly acidic environment such as the human gastrointestinal tract [100]. Martínez-Flores et al. [114] obtained FPC from defatted and dehulled flaxseed after solubilization at pH 11 and precipitation at pH 4.8, containing 66% of protein. This concentrate had the high absorption capacities of oil (1 g/g) and water (2.54 g/g). The functional properties of FPC were affected by pH, whereas its highest emulsifying capacity (84.8%), emulsifying activity (88.4%), and foam stability (83.3%) were observed at a pH of 6.

Like a similar nutritional profile, FPI with various content of mucilage also has comparable functional properties to soy proteins, particularly surface tension, emulsifying activities (50–98% compared to 57% for soy proteins), and water absorption capacity (3.0–6.1 g/g compared to 4.9 g/g for soy proteins). The lower solubility of FPs in water can be increased by heat treatment but at the expense of reduced fat-binding capacity or foaming and emulsifying activities [34]. Waszkowiak and Mikołajczak [115] observed the changes in the spectrum of FPs fraction during the roasting of flaxseeds between 160 and 200 °C. They report an increase of 17 and 19 kDa and a decrease of 13 kDa protein fractions. Since the 13 kDa proteins have been previously reported as crucial for the functional and biological properties within flaxseed, the authors discuss the possible changes of these properties by roasted flaxseeds compared to untreated seeds. Wei et al. [116] hydrolyzed FPs by commercial proteases Alcalase^®^ and Flavourzyme^®^ to prepare peptides of various MW. These hydrolysates can be processed with xylose and L-cysteine by heat treatment to produce Maillard reaction products of different flavor characteristics. Peptides with MW above 1000 Da generated products improve the mouthfulness and flavor stability in umami soup. The products of low MW peptides (128–1000 Da) had a meat-like flavor along with bitterness and umami taste. Further analyses performed by these authors confirmed the presence of volatile, sulfur- and nitrogen-containing compounds in the Maillard reaction products of low-molecular peptides (<1000 Da). These compounds can lead to a meaty aroma of the obtained products [117].

**Table 5:** Functional properties of flaxseed proteins (including protein hydrolysates) and their potential applications in food products.

Functional Properties	Potential Application and Effects/Types of Food Products	References
Solubility	Fortification of beverages/protein drinks	[110,114]
Foaming activity and stability	Texture improvement of aerated products; egg white substitution/whipped desserts or other similar products	[35,114,118,119]
Emulsifying activity, capacity and emulsion stability	Texture improvement; stabilisation of products; oil dispersion; egg yolk substitution/(meat) emulsions, ice creams, sauces	[35,114,118,119]
Thermal stability	Functional agent in heat-processed food products/thermal-treated food products	[100]
Water holding and oil binding capacities	Improvement of texture, softness and juiciness/meat products, bakery products	[34,100,118,119]
Interactions with (poly)saccharides	Synergically with carbohydrates increase in viscosity and viscoelasticity; water-binding capacity; improvement of emulsion stability, foaming capacity, foam stability; texture improvement; coating of food products for improvement of quality attributes and shelf-life; transport of the encapsulated probiotics into human intestine/meat emulsions, sauces, ice creams, gelled food products, food supplements	[36,75,120,121,122,123,124]
Fungistatic activity	Prolonged shelf-life of food products/short shelf-life food products	[125,126,127]
Sensory characteristics (proteins and thermal-treated protein hydrolysates)	Improvement of sensory properties of food products (color, aroma, flavour)/various food products	[94,116,117]

FP-based products containing various content of FG may also possess some functional properties highly usable in food products. FPC and FPI with a high level of mucilage provide higher viscosity (7.7–8.3 vs. 6.5 mPa·s), water absorption (4.19–4.7 g/g vs. 3.03–3.66 g/g), better emulsifying activity (96–98 vs. 50–51%), emulsion stability (90–94 vs. 72–79%) and foaming capacity 166–226 vs. 10–27%) than FPI and FPC with low mucilage content. However, the fat absorption capacity was lower within FPC and FPI with a high content of FG compared to products with a low content of FG (0.95–1.41 g/g vs. 3.13 g/g) [36]. FPs of the 1–4% amount (*w*/*w*) were blended with mungbean starch to form composite gels with softer textures estimated by a reduced hardness (from 2.4 N for only mungbean starch gel to 1.0 N for a composite gel containing 4% of FPs). These gels can serve as a functional agent in food products suitable for various consumers, especially those with swallowing challenges [122]. A coating agent made from FG and FPs, resistant to an acidic environment, has been successfully used in the spray drying process to encapsulate two probiotic bacteria (*Lactobacillus plantarum* and *Bifidobacterium infantis*) to protect them in the gastrointestinal tract [124].

### 3.4. Biological and Physiological Activities

FPI may have 68% in vitro digestibility, similar to flaxseed, lacking mucilage and oil. Thus, removing these constituents and thermal treatment enhanced flaxseed protein digestibility [128]. While native FPs possess various functional properties, valuable biological activities are provided mainly by hydrolyzed FPs or naturally occurring flaxseed peptides. However, crude FPs extracts may also have valuable biological activities. Due to fungistatic activities, FPs are promising ingredients for food preservation against fungal spoilage. It was observed that the >50% antifungal activity of protein extract (concentration in test media = 0.47%) against fungi *Penicillium* sp., *Fusarium graminearum*, *Aspergillus flavus*, and ≥ 40% activity against *Penicillium chrysogenum* [126,127]. Oomah [129] also suggests a preventive effect of FPs against certain forms of colon cancer due to the high cysteine and methionine content in FPs, which can potentially increase antioxidant levels in the body and stabilize DNA during cell division. FPs are abundant in glutamine and arginine, which are essential in preventing and treating heart diseases and supporting the immune system [1].

The single protein hydrolysate fractions and peptide mixtures of flaxseed protein hydrolysates have the antihypertensive potential via angiotensin-converting enzyme inhibition [103,130]. The ability of these hydrolysates to inhibit the enzyme renin has also been demonstrated [103]. Udenigwe et al. [131] described the scavenging activities of three radicals for two fractions of hydrolyzed FPs prepared using seven proteases, whereas the biological activities of these peptide fractions depended on the catalytic specificity of the proteases, as well as the MW of peptides. Silva et al. [132] reported that hydrophobic amino acids, specifically glycine, phenylalanine, tryptophan, cysteine, and alanine contained within FPs, can contribute to the high antioxidant activity of derived peptides. Furthermore, these authors report the higher antioxidant potential, expressed by higher scavenging activity against ABTS radical of fractions containing predominantly low MW peptides compared to the fractions comprised of larger peptides. Hwang et al. [133] suggested that low-molecular hydrolyzed peptides can exhibit higher scavenging activity expressed by the lower IC_50_ value (10.63 µg/mL) of the 1–3 kDa fraction compared to the other fractions or standard compounds, such as vitamin C (13.89 µg/mL), vitamin E (71.43 µg/mL), β-hydroxy acid (31.25 µg/mL). They also reported the antibacterial activity of <1 kDa peptide fraction against *Pseudomonas aeruginosa* and *Escherichia coli* that increased linearly according to the concentration of peptides (20, 40, and 60 µg/mL) in the sample. The roasting of FM led to the formation of Maillard reaction products due to the interactions of contained proteins with polysaccharides. The roasting of FM from three cultivars of flax slightly increased the antiradical capacity assessed by ORAC_FL Assay and expressed as µmol of Trolox equivalents (TE) per gram of defatted FM at 200 °C (avg. 68.1 µmol TE/1 g FM) compared to the FM treated at 180 °C (avg. 61.5 µmol TE/1 g FM). However, its antiradical activity is still much lower than FM treated at 160 °C (avg. 78.0 µmol TE/1 g FM) or untreated FM (avg. 122.5 µmol TE/1 g FM) [115].

In addition to antihypertensive, antioxidant, and other activities, hydrolysates of FPs have been shown to have anti-diabetic activity [134]. Further properties of flaxseed protein-derived peptides are represented by anti-inflammatory activities [131] and preventive effects against neurodegenerative disorders [135,136].

Flaxseed also contains cyclolinopeptides (CLPs), naturally occurring mainly in cotyledon [137]. The immunosuppressive and antimalarial activities of these peptides were described, as well as their ability to inhibit cholate accumulation in the liver [37]. The antioxidant activity of CLPs has also been reported, where improvement in the oxidative stability of flaxseed oil via CLPs was observed [138]. There were also observed antithrombotic activities of the other naturally occurring low MW protein hirudin [139] and antifungal activity of pathogenesis-related protein linusitin [140].

### 3.5. Potential Applications

Vegetable proteins in the forms of meals, concentrate, and isolates are well suitable for their properties in food products or other applications [12]. A protein-rich FM can partly substitute 10–20% of wheat flour in dough without a loss of texture and swelling reduction during baking. However, dough processing can be negatively affected due to the presence of flaxseed polysaccharides in flaxseed flour [106]. The substitution of wheat flour with ground flaxseed in bread (concentration: 15, 25, and 30%) and muffins (concentration: 33, 50, and 66%) affected the color of the products with the increasing concentration of flaxseed in dough during the Maillard reaction. These bakery products may also exhibit pleasant nutty flavors [141]. In cereals or other extruded products, FM can increase the viscoelastic properties due to the formation of crosslinks within the network between FPs and starch [142].

Flaxseed protein-based ingredients can increase the shelf-life of food products. Semolina blended with 15% (*w*/*w*) FG within refrigerated pasta exhibited prolonged shelf-life (from two weeks up to five weeks) due to its fungistatic effect on the growth of molds [125]. This effect can be explained by the previously described antifungal activities of FPs, which may allow for the use of FPs as a preservative in various food products [126]. FPs are also applicable in protein-based edible coatings. The FP-based coating, containing 3 and 5% (*w*/*v*) FPI, covering whole guavas, improved the quality attributes compared to control samples, e.g., retardation of oxidative browning, prevention of loss of polyphenol content (53% for 5% FP-based coated sample vs. 71% for non-coated sample, after 16 days of storage) and ascorbic acid (38% for 5% FP-based coated sample vs. 44% for non-coated sample, after 16 days of storage) and suppression of microbial spoilage. The retardation of these processes enhanced the shelf-life of fruit by up to 16 days [123]. The FP-based coating containing a 15% concentration of proteins and 9% of the raspberry press cake, enriched with ellagic acid formulation, can improve the color and functionality of foods and be an example of a new generation of functional foods/nutraceuticals [143]. Wang et al. [144] reported the applications of FPC containing FG as a functional agent within the food industry, mainly in meat products or ice creams. In ice creams, FPC and FPI at levels of 0.5 or 1.0% can substitute gelatin. It was observed that both components had a comparable melting time [121]. FPC and FPI, added to the meat emulsion in the content of 2.0% to 3.6% depending on their protein content, showed reduced water, fat, and total cooking loss (15.7–25.6 vs. 31.0%) and firmness (520–737 vs. 1111 kPa) in cooked meat emulsion. They also increased the viscosity, emulsifying activity, and emulsion stability in fish sauce [121]. FP-derived hydrolysates produced from the modified flaxseed flour exhibited a good oxidative-stable, functional, and nutritional ingredient in fortified spreads when they represented approximately 41% of the content of the spread. The total flavonoids and other phenolic content were significantly higher than the other spreadable products [145]. FPI and FPC also exhibit neutral to positive flavours improving their potential usage in the food industry [94]. Consumers’ acceptance of products containing FPs of the appropriate amount, reported in several studies, is a crucial finding allowing for the potential use of FPs in food products [146,147].

FPs share similar nutritional and functional properties with soy protein. However, concerning the fact that the commercial production of FPC is very limited and FPI are even unavailable compared to isolates and concentrates of soy, it is not expectable that FPs will replace the soy, or other popular vegetable proteins from pea and wheat, as the nutrient or functional agent in the food products, especially on the industrial scale. However, we find that FPs might have a great potential within specialized food- and health-related applications, for instance, in the food coatings for their antioxidant and antimicrobial properties or as the functional constituent of polysaccharide–protein coacervates. Furthermore, the high potential might have the hydrolyzed products of FPs in the forms of bioactive peptides and the products of the Maillard reaction. Apart from the properties and potential applications of these products reported in this review, we suppose that further approaches in processing FPs to obtain similar or new types of products and suggestions for their use remain to be discovered.

## 4. Practical Comparison of FG and FPs with other Commonly Used Hydrocolloids

Although this paper primarily focuses on flaxseed hydrocolloids, we find it useful to briefly describe the flaxseed hydrocolloids in the context of other widely used and popular hydrocolloids. One of the most suitable matrices to compare different hydrocolloid properties in a real food matrix is yogurt since hydrocolloids are commonly added to this type of food product [148], and its viscosity is a very important quality parameter [149]. Because viscosity is one of the most important rheological properties of hydrocolloids, we chose it to compare different hydrocolloids added to yogurt. Thus, Table 6 summarizes the effects of different hydrocolloids addition on the apparent viscosity of yogurt and offers a direct comparison of FG and FPs with other hydrocolloids.

The viscosity of yogurt increased after the addition of hydrocolloids in most of the cases, except for bovine gelatin and whey protein, where viscosity was lower compared to control. However, it must be pointed out that different amounts of hydrocolloids were added to yogurt samples within compared studies. If we consider the added amount of hydrocolloid and increment of viscosity, then xanthan gum would have the best ratio of the amount per unit of increment. In this context, FG would be roughly comparable, for example, with pectin. On the other hand, FPs in the form of demucilaged flaxseeds [150] caused only approximately a 25% increase in viscosity; however, 3% of FPs were added. The authors of the study mention that the properties of the material were also affected by the presence of fiber. It is the limitation of the study, and unfortunately, there is no available literature regarding the use of FPs in yogurt. In general, it is very hard to compare the results because not all authors use a unified methodology and the same type of yogurt to describe hydrocolloid properties. However, the table shows a trend that might be generalized. Although viscosity is not the only significant hydrocolloid property, comparing a wider spectrum of parameters of other hydrocolloids with FG and FPs would be beyond the scope of the manuscript.

## 5. Conclusions

As a popular functional food, flaxseed contains constituents with significant biological and physiological activities. Apart from the fatty acids and lignans, flaxseed polysaccharides and proteins are promising sources of these properties. Regarding the specific applications, FG can increase the viscosity of juices, salad dressings, and bakery products. High water and oil binding capacities of FG and FPs can be applied to improve the texture of meat and bakery products, whereas emulsifying properties of both hydrocolloids improve the firmness and elasticity of these products, as well as the stability of oil and water emulsions. As a stabilizing and thickening agent, FG may improve creaming and foaming stabilities, decrease the syneresis rate of yogurts and ice creams, and positively affects the pasting and dough rheology of bakery products. As the foaming agent, FPs can substitute white eggs, for instance, in vegan aerated and whipped products. Along with health-related antioxidant and antimicrobial activities of FG, FPs, and derived peptides, they can be used as potent food preservatives or bioactive components of the food coatings, usable to preserve the microbiological quality and sensory parameters of short shelf-life foods and processed products. Protective and growth-promoting capacities towards some probiotics allow the potential application of FG as a prebiotic. FG can also be potentially useful in treating gastrointestinal and cardiovascular diseases. FG shares most functional properties on a comparable scale with other plant gums, such as guar, arabic, tragacanth guar gums, and carrageenan. Thus, it is possible to substitute them effectively in most food products, where these gums serve as important additives. Compared to some of the other plant gums, FG is easily extractable by water from the whole seeds of flax, which can be potentially more accessible compared to sources of gums produced from saps or algae. Apart from the comparable functioning, the beneficial biological properties allow FG to outclass other gums due to its wider applicability and the extra effects related to these properties in the various products. Flaxseed peptides, naturally occurring or as the products of hydrolyzed FPs, may have health-promoting properties and can be potentially used to treat cardiovascular-related problems, microbial infections, cancer, and as preventive agents against neurodegenerative disorders predetermining their potential use as novel pharmaceuticals. Some low-molecular peptides, in the form of the Maillard reaction products produced by processing of FPs hydrolysates, exhibit a distinct aroma and taste. Thus they could be used as flavoring agents in food products.

Based on the information summarized in this review, flaxseed is a very complex and heterogeneous matrix. Various inner and outer factors determine the composition, yield, and properties of flaxseed polysaccharides and proteins. Thus, to preserve the properties and maximize the potential of flaxseed hydrocolloids, the suitable cultivars as the source of flaxseed and the optimal method for the extraction and processing of FG and FPs need to be selected according to the purpose of their use. FP/FG-based agents, ingredients, and other products, mainly in the food industry or medicine. Although many studies related to FG and FPs have already been published, there has not been any significant development and application of these flaxseed-based products within industrial practices. This fact may be caused by many factors such as the unavailability of the sources of flaxseed due to various limitations in flax growing, the unavailability of industrial technologies for obtaining and processing FG and FPs, or the novelty of the related research. As one of the major issues related to the limited use of flaxseed-based products, we find the fact that flaxseed by-products obtained after oil production are mostly fed by animals instead of using them to fortify the food products or being processed for the extraction of gums and proteins. From our point of view, a need to increase the awareness and popularity in the eyes of the public and stakeholders is crucial, and it is our suggested approach for increasing the use of flaxseed hydrocolloids on an industrial scale for all relevant applications. However, further studies related to the use of flaxseed hydrocolloids within food products, as well as health-related and non-food applications, are still highly needed to be performed.

## Figures and Tables

**Table 4:** Amino acid composition of flaxseed from three cultivars and one subspecies.

Amino Acid	Content (g/100 g Protein)			
	*NorLin* (Brown) [6]	subsp. *panambi* (Brown) [105]	*Omega* (Yellow) [6]	*Foster* (Yellow) [34]
Alanine	4.4	3.8	4.5	4.7
Arginine	9.2	9.4	9.4	10.0
Aspartic acid	9.3	9.9	9.7	10.0
Cysteine	1.1	1.0	1.1	1.8
Glutamic acid	19.6	19.5	19.7	20.0
Glycine	5.8	5.9	5.8	5.9
Histidine ^E^	2.2	2.4	2.3	2.1
Isoleucine ^E^	4.0	3.9	4.0	4.1
Leucine ^E^	5.8	5.7	5.9	6.0
Lysine ^E^	4.0	3.8	3.9	4.0
Methionine ^E^	1.5	1.7	1.4	1.4
Phenylalanine ^E^	4.6	4.8	4.7	4.8
Proline	3.5	3.7	3.5	3.8
Serine	4.5	5.0	4.6	4.7
Threonine ^E^	3.6	4.1	3.7	3.8
Tryptophan ^E^	1.8	1.5	NR	NR
Tyrosine	2.3	2.3	2.3	2.4
Valine ^E^	4.6	4.8	4.7	5.1

^E^: essential amino acid; NR: not reported.

**Table 6:** Comparison of flaxseed gum and protein with other hydrocolloids as affecting apparent viscosity of yogurt.

Hydrocolloid Added to Yogurt	Description of Yogurt	Amount Added (%)	Increase/Decrease in Apparent Viscosity Compared to Control (Pa·s)	Reference
Demucilaged flaxseeds (protein)	The authors did not specify fat and protein content in the yogurt	3.0%	Viscosity ↑ from ca. 1.95 to 2.5	[150]
Flaxseed gum/mucilage	Semi-fat (1.5%) yogurt	0.10, 0.15 and 0.20%	Viscosity ↑ from ca. 2.45 * to 2.90, 3.05 and 3.15, respectively	[151]
κ-Carrageenan	Pot-set yogurt containing 0.1% fat and 3.9% protein	0.01, 0.04, 0.08%	viscosity ↑ from 0.71 * to 0.87, 1.21 and 3.54, respectively	[152]
Starch (modified) ⁑	Pot-set yogurt containing 0.1% fat and 3.9% protein	0.5, 1.0, 1.5%	Change of viscosity from 0.71 * to 0.56, 0.72 and 0.82, respectively, however it was not significant	[152]
Xanthan gum	Pot-set yogurt containing 0.1% fat and 3.9% protein	0.005, 0.01, 0.015%	Viscosity ↑ from 0.71 * to 1.50, 2.79 and 4.35, respectively	[152]
Pectin	Skim yogurt with 0.1% fat	0.20, 0.25, 0.30%	Viscosity ↑ from 0.16 * to 0.38, 0.51 and 0.57, respectively	[153]
Inulin	Skim yogurt with 0.1% fat	7, 8, 9%	Viscosity did not differ (0.16 for all samples)	[153]
Whey protein	Five commercially available whey protein concentrates were compared to skim milk powder * used for yogurt preparation	All yogurts were standardized to 4.5% protein	Viscosity ↓ from 1.10 * to 0.38–84	[154]
Bovine gelatine (140 bloom)	Pot-set yogurt containing 0.1% fat and 3.9% protein	0.5, 1.0, 1.5%	Viscosity ↓ from 0.71 * to 0.29, 0.31 and 0.21, respectively	[152]
Sodium caseinate	Commercially available sodium caseinate compared to skim milk powder * used for yogurt preparation	All yogurts were standardized to 4.5% protein	Viscosity ↑ from ca. 0.9 * to 2	[155]
Soy protein hydrolysates	The authors did not specify fat and protein content in the yogurt	0.1, 0.2, 0.3%	Viscosity changed from ca. 2.11 * to 1.90, 1.99 and 2.2 respectively	[156]

↑: increase; ↓: decrease; *: value assigned with an asterisk belongs to control group; ⁑: hydroxypropyl starch phosphate derived from waxy maize.
